# Erythroid-Specific Expression of *LIN28A* Is Sufficient for Robust Gamma-Globin Gene and Protein Expression in Adult Erythroblasts

**DOI:** 10.1371/journal.pone.0144977

**Published:** 2015-12-16

**Authors:** Y. Terry Lee, Jaira F. de Vasconcellos, Colleen Byrnes, Megha Kaushal, Antoinette Rabel, Laxminath Tumburu, Joshua M. Allwardt, Jeffery L. Miller

**Affiliations:** Molecular Genomics and Therapeutics Section, Molecular Medicine Branch, National Institute of Diabetes and Digestive and Kidney Diseases, National Institutes of Health, Bethesda, Maryland, United States of America; Southern Illinois University School of Medicine, UNITED STATES

## Abstract

Increasing fetal hemoglobin (HbF) levels in adult humans remains an active area in hematologic research. Here we explored erythroid-specific *LIN28A* expression for its effect in regulating *gamma-globin* gene expression and HbF levels in cultured adult erythroblasts. For this purpose, lentiviral transduction vectors were produced with *LIN28A* expression driven by erythroid-specific gene promoter regions of the human *KLF1* or *SPTA1* genes. Transgene expression of *LIN28A* with a linked puromycin resistance marker was restricted to the erythroid lineage as demonstrated by selective survival of erythroid colonies (greater than 95% of all colonies). Erythroblast *LIN28A* over-expression (*LIN28A*-OE) did not significantly affect proliferation or inhibit differentiation. Greater than 70% suppression of total *let-7* microRNA levels was confirmed in *LIN28A*-OE cells. Increases in *gamma-globin* mRNA and protein expression with HbF levels reaching 30–40% were achieved. These data suggest that erythroblast targeting of *LIN28A* expression is sufficient for increasing fetal hemoglobin expression in adult human erythroblasts.

## Introduction

Development consists of a series of orchestrated stage-specific events controlled in both space and time by multiple factors including a network of heterochronic genes. Extensive research performed in the nematode *C*. *elegans* identified several factors involved in early embryonic development, including the RNA binding protein named *lin-28* and its main microRNA (miRNA) target, *let-7*. Mutations in *C*. *elegans lin-28* cause precocious development during larval growth, while loss of *let-7* results in recapitulation of larval cell fates in adult worms [1].

The *LIN28*/*let-7* regulatory pathway remains exquisitely well conserved throughout vertebrate evolution. The sequences of the mature *let-7* miRNAs are identical in most animal species including human. During ontogeny, loss of *LIN28* expression results in a concomitant increase in *let-7* miRNAs in most tissues. In association with *OCT4*, *SOX2* and *NANOG*, *LIN28* reprograms human somatic cells to become pluripotent cells with characteristics of embryonic stem cells [2]. In embryonic and cancer stem cells, *LIN28* enhances proliferation and self-renewal [3]. In contrast to stem cells, reduced expression of *let-7* miRNAs in nonmalignant muscle cells and hepatocytes enhances differentiation of the cells [4, 5]. Genetic manipulation of *Lin28*/*let-7* in mice also regulates glucose metabolism [6]. Since the phenotypic effects of *let-7* expression are highly dependent upon the transcriptome of the cell in which it is expressed, *LIN28* is thus predicted to be functionally pleomorphic with tissue- and cell-type specificity.

The expression of human *LIN28* genes has been associated with variations in body stature and timing of puberty [7–10]. The two known human homolog genes of the *C*. *elegans lin-28* are *LIN28A* and *LIN28B*. In human CD34(+) cells, expression of *LIN28A* or *LIN28B* in culture causes increased expression of *gamma-globin* in conjunction with erythroid differentiation [11, 12]. However, it is unclear whether LIN28 reprograms CD34(+) stem cells, or alternatively, LIN28 acts directly among committed erythroblasts to increase the expression of the *gamma-globin* genes. To address this topic, we explored the effects of erythroid-targeted LIN28 expression in cultured hematopoietic cells from healthy adult humans.

## Materials and Methods

### Ethics Statement

Approval for the research protocol and consent documents pertaining to all studies using primary erythroblasts was granted by the National Institute of Diabetes and Digestive and Kidney Diseases Institutional Review Board. Written informed consent was obtained from all research subjects prior to participation in this study.

### Cell culture

Cryopreserved healthy adult human CD34(+) cells were cultured *ex vivo* in a 3-week serum-free system consisting of three phases: phase I from day 0 to 7; phase II from day 7 to 14; and phase III from day 14 to 21 as previously described [11].

### Lentiviral erythroid promoter vector construction

Lentiviral backbone pLVX-IRES-Puro (Cat. 632183) was purchased from Clontech (Mountain View, CA) for construction of the *KLF1* promoter and *SPTA1* promoter *LIN28A* over-expression (OE) vector. The *LIN28A* coding region with added XhoI and NotI restriction sites for directional cloning was synthesized by Eurofins MWG Operon Inc. (Huntsville, AL). The synthetic *LIN28A* coding region was digested with XhoI and NotI restriction enzymes (New England Biolabs, Ipswich, MA) following manufacturer’s protocol and cleaned up with MinElute Reaction Cleanup Kit (Qiagen, Valencia, CA), followed by cloning into the pLVX-IRES-Puro vector to generate a pLVX-LIN28A-IRES-Puro plasmid. To generate the KLF1-LIN28A-IRES-Puro (KLF1-LIN28A-OE) plasmid, the CMV promoter from the pLVX-LIN28A-IRES-Puro was replaced with the human *KLF1* promoter by directional cloning with ClaI and XhoI restriction enzymes. The *KLF1* promoter was PCR amplified from human genomic DNA using the following PCR primer pairs: 5’KLF1 primer: 5’-AAATCGATGGTACCGGCTGGTCTTGAAATCCTGGTGTCAA-3’; primer: 5’- ACTCGAGTGGCTGGCTGGTGCCCACCCTGGGCCTC-3’ using CloneAmp HiFi PCR Premix (Clontech). SPTA1-LIN28A-IRES-Puro (SPTA1-LIN28A-OE) was constructed by replacing the *KLF1* promoter from KLF1-LIN28A-IRES-Puro plasmid with the human *SPTA1* promoter by directional cloning with ClaI and XhoI restriction enzymes. *SPTA1* promoter PCR primers were modified from previous reports [13, 14]. The *SPTA1* promoter was PCR amplified from human genomic DNA with the following primers: 5’SPTA1 primer: 5’- GCCATCGATGGTACCAGACTTTCAAGAAGAGAATGT-3’ and 3’SPTA1 primer: 5’-AACTCGAGGGTTTAGAACCTGGCAAGATAA-3’ using CloneAmp HiFi PCR Premix (Clontech). Empty control vectors containing the KLF1 and SPTA1 promoters were constructed by replacing the CMV promoter in pLVX-IRES-Puro vector with the KLF1 or SPTA1 promoter using the same restriction cloning strategy. The KLF1 and SPTA1 promoter sequences used for these constructs are shown in the S1 File.

### Virus production

For lentivirus production, HEK293T cells (Thermo Scientific, Waltham, MA) were plated in 100-mm poly-l-lysine coated plates (BD Biosciences, San Jose, CA) with DMEM complete media (containing 10% FBS, l-glutamine and penicillin-streptomycin) (Life Technologies, Grand Island, NY). The plasmid mixture was prepared for co-transfection following the manufacturer’s protocol for the Calcium Phosphate Transfection Kit (Life Technologies). The co-transfection mixture consists of the vector plasmid (empty vector control, KLF1-LIN28A-OE or SPTA1-LIN28A-OE vector) with packaging helper virus plasmids [15] as follows: CAG kGP1.1R, CAG4 RTR2, and CAGGS vsv-g (generously provided by Drs. Derek Persons and Arthur Nienhuis, St. Jude Children’s Hospital, Memphis, TN) [16]. The day after transfection, the media was changed to DMEM with l-glutamine and penicillin-streptomycin without FBS for virus production. The lentivirus-containing supernatant was concentrated overnight following the Lenti-X Concentrator (Clontech) manufacturer’s protocol and resuspended in 1/100 phase I culture medium of the original supernatant volume. Viral titer estimates were determined using the Lenti-X GoStix (Clontech) following the manufacturer’s instructions. A MOI of 5 was calculated for the viral transductions.

### Lentiviral Transduction

Cryopreserved CD34(+) cells were thawed and seeded at a concentration of 250,000 cells/ml in phase I culture medium. On day 3, 300,000 cells were resuspended at 2,000 cells/μl in phase I culture medium and transduced with viral particles. After 24 hours, the cells were resuspended in 4.0 ml phase I culture medium containing puromycin and transferred on day 7 into phase II culture medium without puromycin at 20,000 cells/ml. For each transduction, a puromycin selection control of mock-transduced cells was included until the end of phase II culture and analyzed by flow cytometry to confirm puromycin selection.

### Colony Formation Assay

CD34(+) cells from three donors were transduced with KLF1-LIN28A-OE or SPTA1-LIN28A-OE vectors overnight and then mixed in MethoCult H4034 Optimum media (Stem Cell Technologies, Vancouver, Canada) supplemented with puromycin for colony formation assay with duplicate wells following manufacturer’s protocol. CMV-LIN28A-OE lentiviral particles [12] were performed for comparison. On culture day 14, colonies of erythroid progenitors (BFU-E), granulocyte-macrophage progenitors (CFU-GM, CFU-G and CFU-M) and multi-potential granulocyte, erythroid, macrophage, megakaryocyte progenitors (CFU-GEMM) were enumerated from each donor.

### Flow Cytometry Analyses

On culture days 14 and 21, cells were stained with CD71 antibody, clone T56/14, R-PE (phycoerythrin) conjugate (Invitrogen, Carlsbad, CA) and glycophorin A (GPA) antibody, clone CLB-ery-1 fluorescein (FITC) conjugate (Invitrogen) and cell differentiation was assessed using the BD FACSAria I flow cytometer (BD Biosciences) as previously described [17]. A minimum of 5,000 live cell events was recorded and positively stained cell populations that had a fluorescence signal above two standard deviations were defined as positive.

### Quantitative PCR analysis

Q-RT-PCR assays and conditions were performed as previously described [11, 18, 19].

### *Let-7* family of miRNAs quantitative PCR analysis

Absolute quantification for each *let-7* family member was determined by constructing a standard curve prepared on the basis of the respectively synthetic targeted mature miRNA oligonucleotide of known concentration (1:10 serial dilutions, n = 6) that was run in parallel with biological samples. Each reaction was performed in triplicate. Complementary DNA and real-time PCR reaction using Taqman microRNA assay (Applied Biosystems, Grand Island, NY) were performed as previously described [20] for *let-7a*, *let-7b*, *let-7c*, *let-7d*, *let-7e*, *let-7f-2*, *let-7g*, *let-7i* and *miR-98*.

### HPLC analysis of fetal and adult hemoglobin

Two million cultured cells at day 21 were pelleted, resuspended in distilled water and further lysed by two cycles of repeated freeze-thaw in a dry ice ethanol bath. Cell debris was removed by filtration through Ultrafree-MC devices (Millipore, Billerica, MA). Hemoglobin content was analyzed for HbF and HbA using a 20x4 mm PolyCATA column (Poly LC, Columbia, MD) fitted to a Gilson HPLC system (Gilson, Middleton, WI) as previously described [21, 22]. The adult globin peak (HbA) and fetal globin peak (HbF) were quantitated and compared using Gilson Unipoint LC software (version 5.11). Total areas under the HbA and HbF peaks were used for ratio comparisons.

### Statistical analysis

Replicate data are expressed as mean value ± SD with significance calculated by two-tailed Student’s t test.

## Results

### *LIN28A* transcription driven by KLF1 or SPTA1 promoter results in erythroid-specific expression

**Fig 1:**
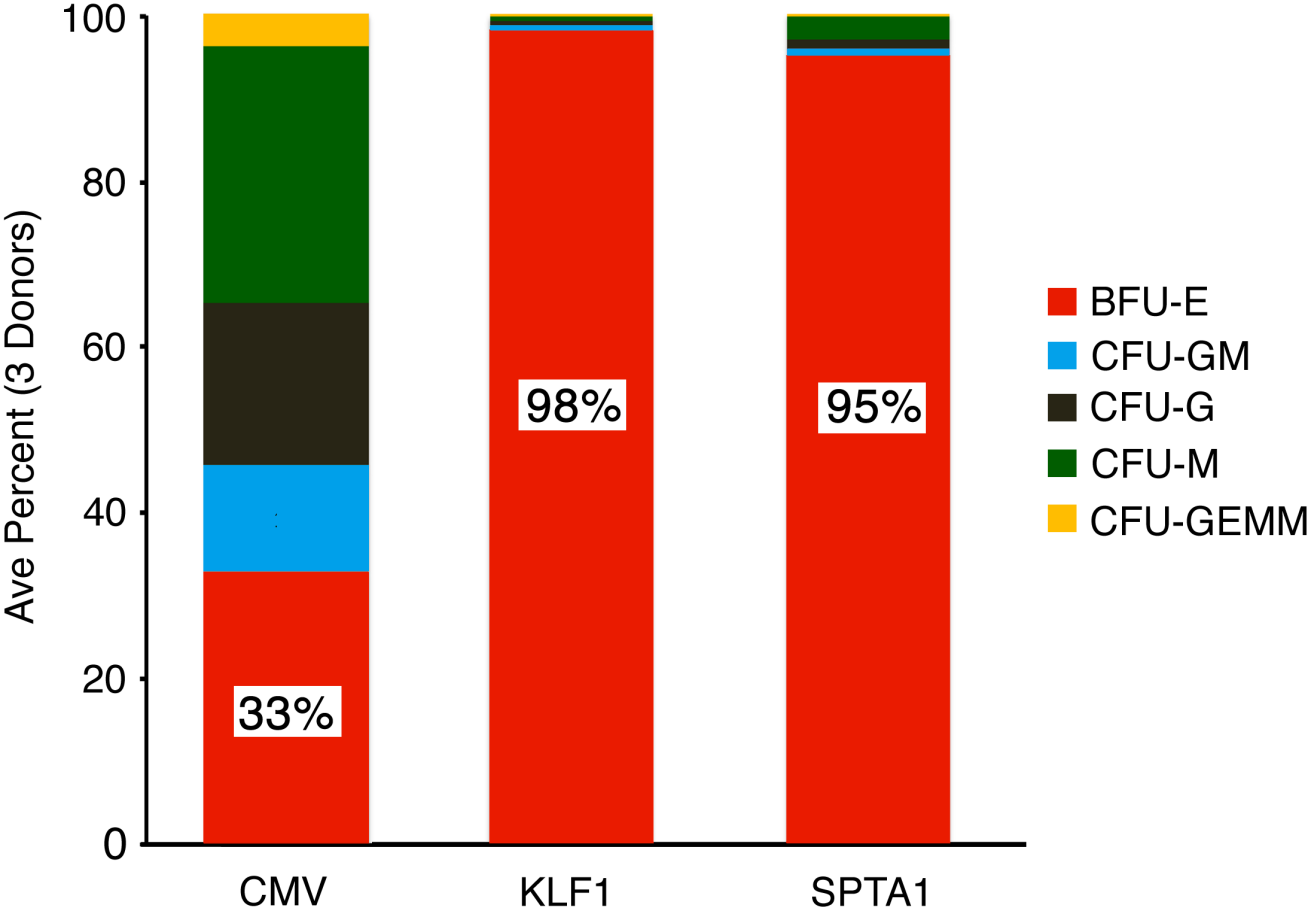
Erythroid-specific over-expression of *LIN28A* confirmed by colony formation assays. Cells were transduced with lentivirus particles for *LIN28A* over-expression driven by a CMV, KLF1, or SPTA1 promoter (See Methods). Transduced cells were cultured for 14 days in semi-solid methylcellulose medium supplemented with puromycin. Average colony counts were obtained from duplicate wells of each condition (three separate donors). The average percentage of each colony type is shown as separate colors (BFU-E percentage shown in red bar field; color key on the right).

For erythroid expression of *LIN28A*, a lentiviral vector backbone with erythroid-specific expression of LIN28A was designed using the promoter region of the human erythroid genes Kruppel-like factor 1 (*KLF1*) [Prof. James J. Bieker, Mount Sinai School of Medicine, New York, NY, personal communication] or spectrin alpha chain erythrocytic 1 (*SPTA1*) [13, 14]. The vector backbone features an internal ribosome entry site (IRES) from the encephalomyocarditis virus (EMCV) positioned after the *LIN28A* coding region to facilitate cap-independent translation of the puromycin resistant gene, while expression of the bicistronic transcript remained driven by the erythroid-specific promoter KLF1 or SPTA1. To investigate erythroid specificity, a colony formation assay was performed in adult CD34(+) cells treated with *LIN28A* over-expression lentivirus with the KLF1 promoter (KLF1-LIN28A-OE) or the SPTA1 promoter (SPTA1-LIN28A-OE). As shown in Fig 1, the erythroid specific expression of these vectors was demonstrated by erythroblast survival with less than 5% of puromycin-resistant colonies being non-erythroid (BFU-E: KLF1-LIN28A-OE: 98.4 ± 0.7%; SPTA1-LIN28A-OE: 95.2 ± 1.1%). By comparison, expression of LIN28A-Puro under a constitutively active CMV promoter resulted in greater than 50% non-erythroid puromycin-resistant colony formation (CMV-LIN28A-OE: BFU-E: 32.9 ± 3.0%; CFU-GM: 12.9 ± 1.5%; CFU-G: 19.4 ± 5.7%; CFU-M: 30.7 ± 6.5%; GEMM: 4.0 ± 2.5%; Fig 1).

**Fig 2:**
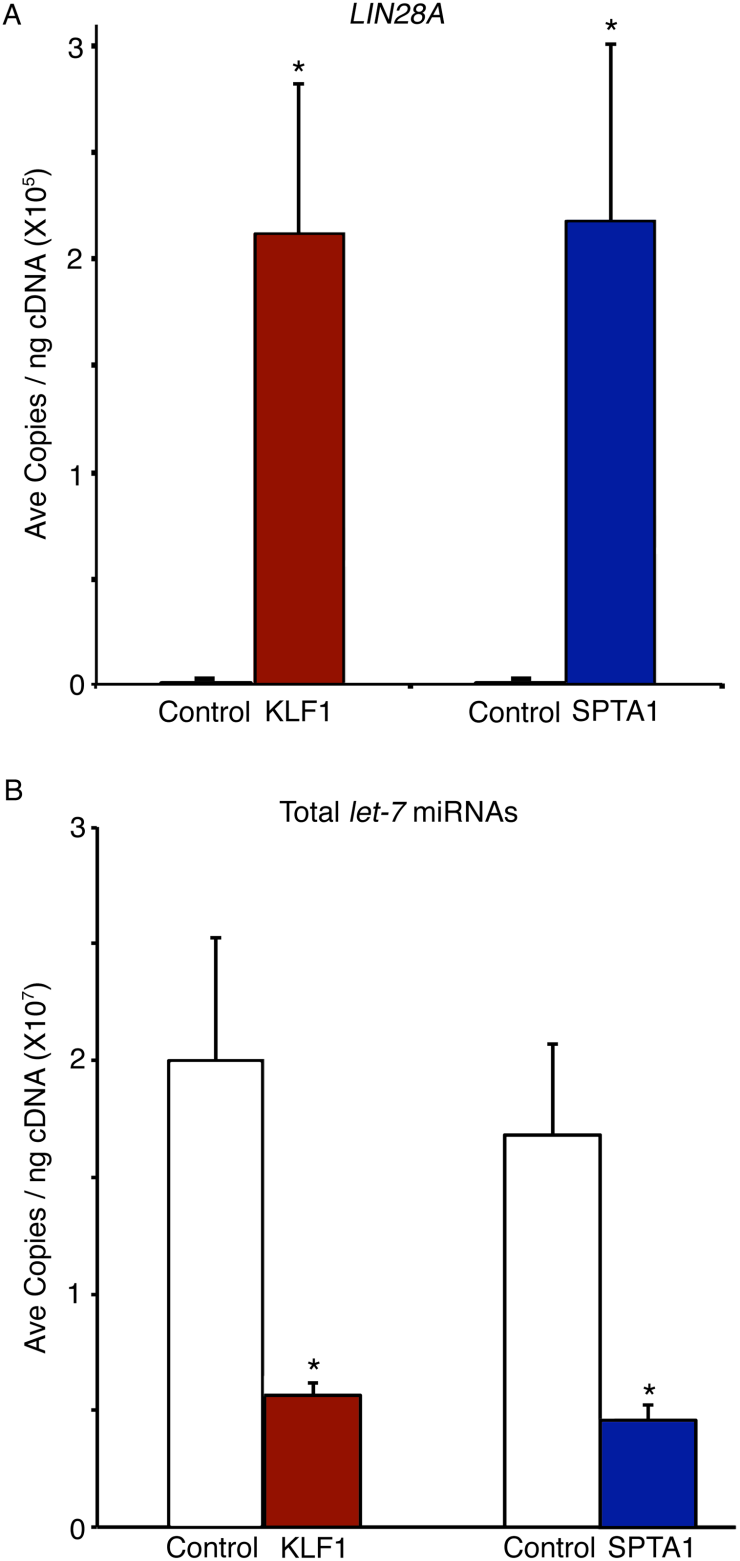
*LIN28A* over-expression mediated by KLF1 or SPTA1 promoter regulates the *let-7* family of miRNAs. RNA samples from erythroblasts cultured on day 14 were examined for **(A)** *LIN28A* over-expression and **(B)** the total levels of *let-7* miRNAs using Q-RT-PCR. Mean value ± SD from three separate donors for each condition: KLF1-Empty vector control (control, open bar), KLF1-LIN28A-OE (KLF1, red bar), SPTA1-Empty vector control (control, open bar), and SPTA1-LIN28A-OE (SPTA1, blue bar). Asterisks indicate p<0.05.

In addition to puromycin resistance, *LIN28A*-OE was confirmed by Q-RT-PCR analysis at culture day 14 [Fig 2A; KLF1-Empty and SPTA1-Empty vector controls: below detection limits; KLF1-LIN28A-OE: 2.1E+05 ± 7.0E+04 copies/ng; SPTA1-LIN28A-OE: 2.2E+05 ± 8.3E+04 copies/ng; p<0.05]. LIN28 proteins are known regulators of the *let-7* family of miRNAs [3, 23–25], and over-expression of LIN28 in CD34(+) cells from healthy volunteers has been shown to strongly down-regulate several *let-7* family members [11]. To determine if *LIN28A*-OE driven by KLF1 or SPTA1 promoters produced a functional protein, KLF1-LIN28A-OE and SPTA1-LIN28A-OE samples were investigated for the expression of *let-7* miRNAs. As shown in Fig 2B, the total levels of *let-7* miRNAs in the *LIN28A*-OE samples with KLF1 or SPTA1 promoters was significantly down-regulated when compared to the respective control transductions [KLF1-Empty vector control: 2.0E+07 ± 5.3E+06 copies/ng; KLF1-LIN28A-OE: 5.6E+06 ± 5.6E+05 copies/ng, p = 0.046; SPTA1-Empty vector control: 1.7E+07 ± 3.9E+06; SPTA1-LIN28A-OE: 4.6E+06 ± 6.2E+05 copies/ng, p = 0.040]. Altogether, these results demonstrate that erythroid-specific *LIN28A*-OE produces a functional LIN28A protein capable of erythroid suppression of the *let-7* family of miRNAs.

### Erythroid *LIN28A* does not affect cell proliferation or terminal maturation of cultured erythroblasts

**Fig 3:**
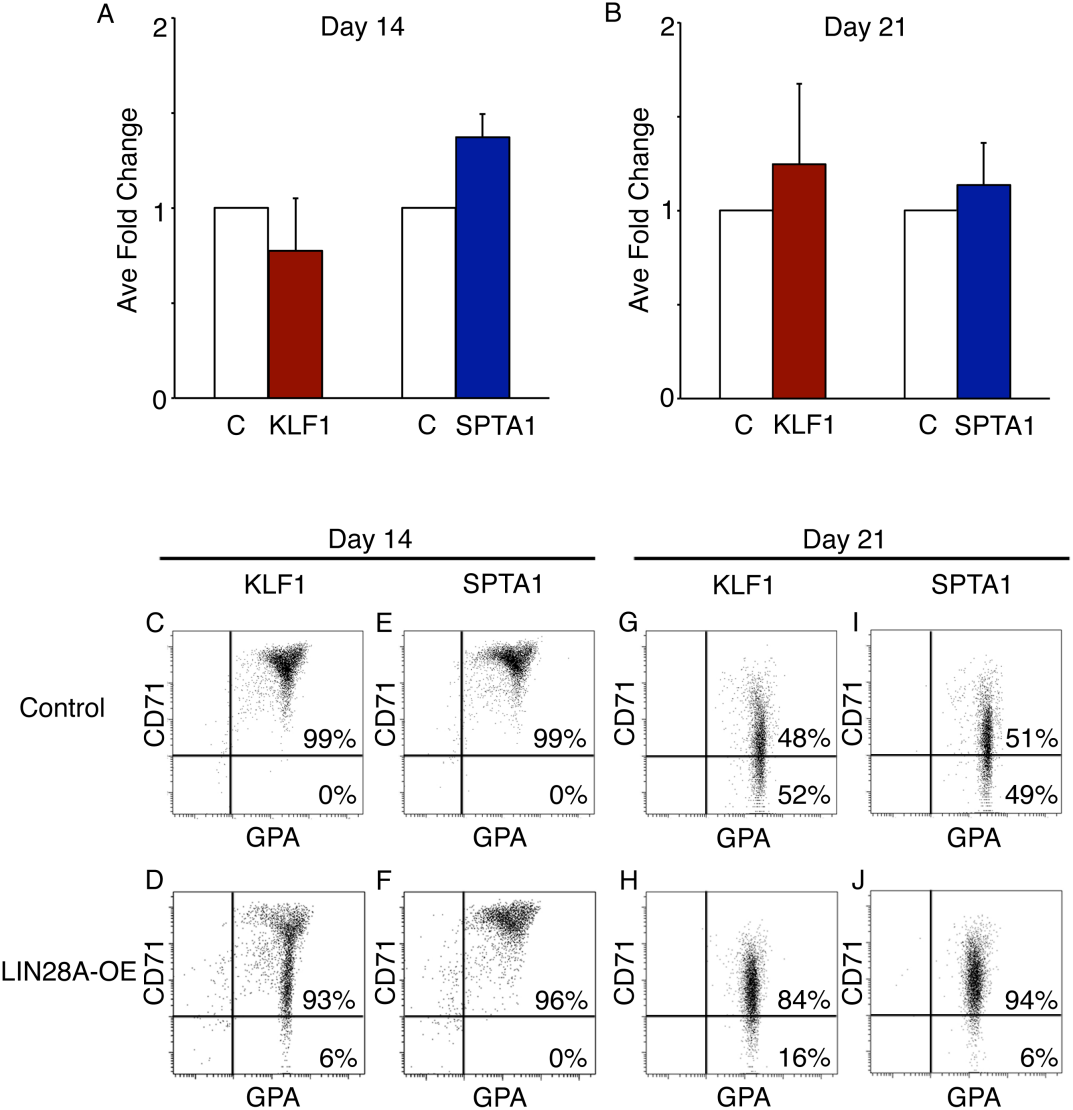
*LIN28A* erythroid-specific over-expression does not affect cell proliferation or prevent terminal maturation of cultured erythroblasts. Cell proliferation was assessed by cell counts performed on **(A)** culture day 14 and **(B)** culture day 21. Mean fold change ± SD from three separate donors for each condition: KLF1-Empty vector control (C, open bar), KLF1-LIN28A-OE (KLF1, red bar), SPTA1-Empty vector control (C, open bar), and SPTA1-LIN28A-OE (SPTA1, blue bar). Representative flow cytometry dot plots of cells stained with antibodies against transferrin receptor (CD71) and glycophorin A (GPA) cultured on **(C-F)** day 14 and **(G-J)** day 21 with percentages shown. KLF1-Empty vector control (control, panels C and G), KLF1-LIN28A-OE (KLF1, panels D and H), SPTA1-Empty vector control (control, panels E and I), and SPTA1-LIN28A-OE (SPTA1, panels F and J).

To evaluate the effects of *LIN28A*-OE in cell proliferation, the cell counts on culture days 14 and 21 were compared between KLF1-LIN28A-OE, SPTA1-LIN28A-OE and each respective empty vector control. No significant differences in cell proliferation were observed between the treatments when compared to control samples (Fig 3A and 3B). Erythroblast differentiation was compared between controls and *LIN28A*-OE cells. Flow cytometry analysis of transferrin receptor (CD71) and glycophorin A (GPA) were performed at culture day 14 (Fig 3C–3F) and day 21 (Fig 3G–3J) to determine the levels of erythroblast maturation. Interestingly, on culture day 14 of differentiation, there was a predominant population of high CD71(+) and GPA(+) cells observed in all conditions, but an accelerated maturation was observed in the KLF1-LIN28A-OE samples as demonstrated by decreased levels of CD71 among the GPA(+) cells (compare Fig 3C–3F). On culture day 21, cell maturation was observed at comparable levels in controls, KLF1-LIN28A-OE and SPTA1-LIN28A-OE cells (compare Fig 3G–3J).

### Adult erythroblast *LIN28A* over-expression increases fetal globin mRNA and protein levels

**Fig 4:**
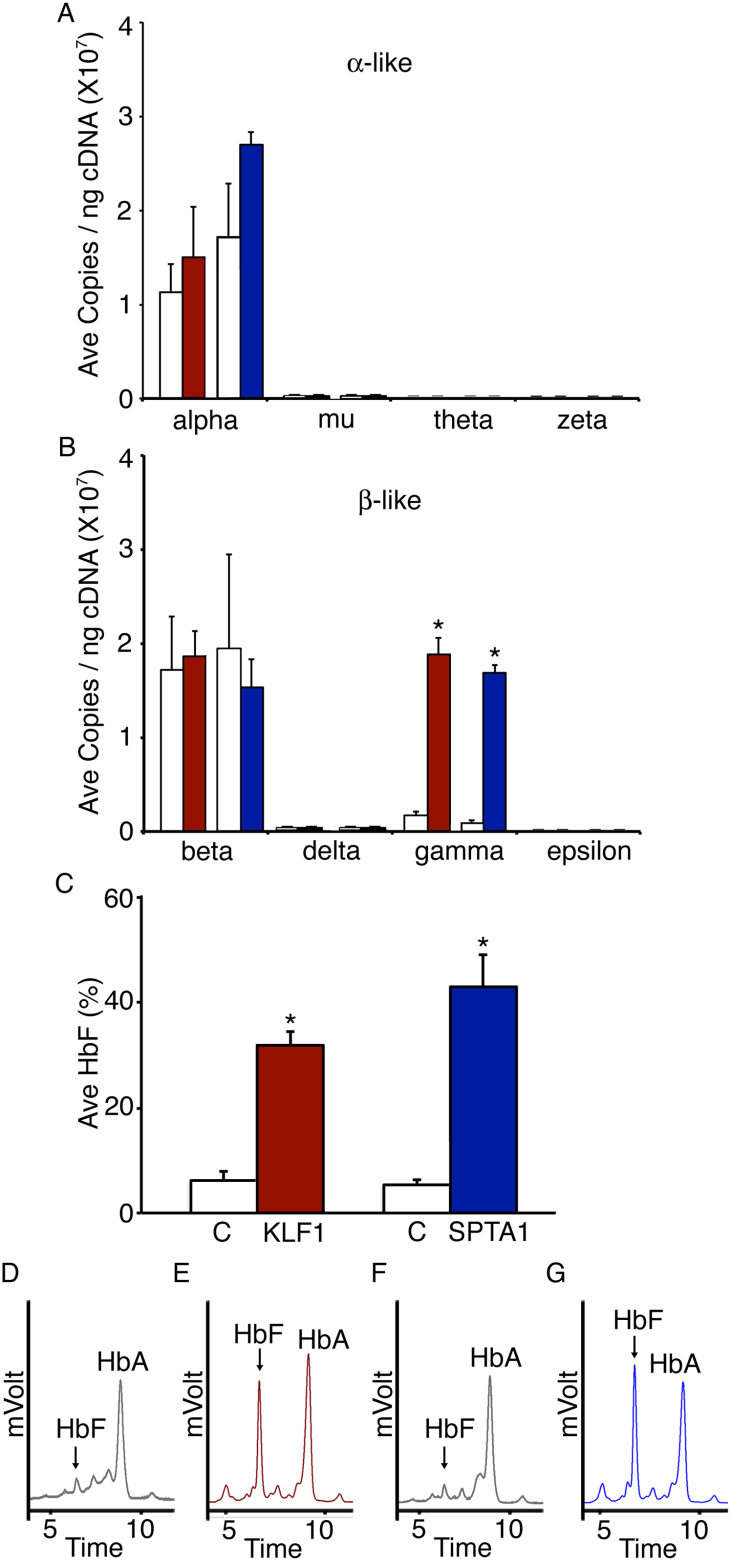
Erythroid-specific *LIN28A* over-expression effects upon globin gene and protein levels in cultured adult erythroblasts. *LIN28A* over-expression driven by KLF1 or SPTA1 promoter compared to control samples in the mRNA expression levels of **(A)** *alpha*-, *mu*-, *theta*- and *zeta*-*globins* and **(B)** *beta*-, *delta*-, *gamma*- and *epsilon*-*globins*. Analyses were performed at culture day 14. **(C)** HPLC analysis of hemoglobin from each respective control, KLF1-LIN28A-OE, and SPTA1-LIN28A-OE erythroblasts at culture day 21. KLF1-Empty vector control (C, open bar), KLF1-LIN28A-OE (KLF1, red bar), SPTA1-Empty vector control (C, open bar), and SPTA1-LIN28A-OE (SPTA1, blue bar). Representative HPLC tracing from **(D)** KLF1-Empty vector control (gray tracing), **(E)** KLF1-LIN28A-OE (red tracing), **(F)** SPTA1-Empty vector control (gray tracing), and **(G)** SPTA1-LIN28A-OE (blue tracing). Mean value ± SD three separate donors for each condition. Asterisks indicate p<0.05.

The effects of erythroid-specific *LIN28A*-OE on the mRNA expression levels of the globin genes was investigated on culture day 14. In the *alpha-globin* locus, no significant changes were observed in the expression levels of *alpha*-, *mu*-, *theta*- or *zeta*-*globin* (Fig 4A) when compared to each respective empty vector control. In the *beta-globin* locus, *beta*-, *delta*- and *epsilon-globin* also demonstrated no major changes (Fig 4B). The slight increase in the levels of *beta-globin* mRNA in KLF1-LIN28A-OE samples may be due to the observed increase in cellular maturation. Remarkably, *gamma-globin* mRNA levels were significantly increased in both KLF1-LIN28A-OE and SPTA1-LIN28A-OE cells when compared to the respective empty vector controls (Fig 4B; KLF1-Empty vector control: 1.7E+06 ± 3.9E+05 copies/ng; KLF1-LIN28A-OE: 1.9E+07 ± 1.7E+06 copies/ng, p = 0.002; SPTA1-Empty vector control: 9.2E+05 ± 2.9E+05 copies/ng; SPTA1-LIN28A-OE: 1.7E+07 ± 8.9E+05 copies/ng, p = 0.001).

In accordance with increased levels of *gamma-globin* mRNA, HbF levels were significantly increased in KLF1-LIN28A-OE and SPTA1-LIN28A-OE when compared to control transductions (Fig 4C–4G; KLF1-Empty vector control: 6.2 ± 1.9%; KLF1-LIN28A-OE: 31.9 ± 2.7%, p = 0.001; SPTA1- Empty vector control: 5.3 ± 1.1%; SPTA1-LIN28A-OE: 43.0 ± 6.2%, p = 0.006).

## Discussion

Highly conserved across evolution, the LIN28 RNA-binding proteins are expressed in the early stages of development and are generally subjected to down-regulation during ontogeny [26]. The RNA-binding mechanism of action for LIN28 proteins is highly directed by recognition of the conserved RNA quadruplet GGAG-motif, which binds to the pri- or pre-*let-7* as well as to several other RNAs throughout the cellular transcriptome [27, 28]. In humans, a defined pattern of the *let-7* miRNA expression during ontogeny is clearly observed in the erythroid lineage throughout the fetal-to-adult transition with significant increased expression of the *let-7* miRNAs in adult cells [20]. Additional data support the notion that the *LIN28*/*let-7* axis is involved in fetal hemoglobin regulation as part of the developmental switching phenomenon [11].

In this study, we demonstrate that erythroid targeted over-expression of *LIN28A* is sufficient for robust increases in *gamma-globin* mRNA and HbF expression in adult human erythroid cells grown *ex vivo*. Lentiviral transduction vectors produced with *LIN28A* expression driven by the promoter region of the human erythroid *KLF1* or *SPTA1* genes were utilized to transduce human CD34(+) cells from adult healthy volunteers. The *KLF1* gene is a transcription factor that is expressed in both primitive and definitive erythroid cell populations [29]. Functionally, *KLF1* binds to several erythroid-specific gene regulatory regions, including the globin gene clusters [30, 31]. The *KLF1* gene promoter was chosen for this study because it is predicted to increase *LIN28* expression in erythroid cells prior to the cells exhibiting high-level globin gene expression. The *SPTA1* gene encodes the alpha subunit of the erythroid spectrin protein, a major component of the red cell membrane skeleton, which is essential for the erythrocyte’s biconcave disk shape and deformability [32–34]. *SPTA1* was chosen for this study because it is exclusively found in the erythroid portion of bone marrow cells [35]. According to our colony formation assays, both *KLF1* and *SPTA1* promoters showed LIN28A expression with puromycin resistance almost exclusively in the cultured erythroblasts.

With both vectors, the expression of LIN28 caused increased expression of the gamma-globin gene and protein. In contrast to the reported effects of LIN28 in stem cells [2, 36], we found that expression of LIN28 in erythroblasts neither caused increased growth nor inhibited maturation. The observed increase in maturation on day 14 of differentiation in the KLF1-driven LIN28 over-expression samples remains unexplained. In contrast to its role in promoting stem cell self-renewal, our data suggest that erythroblast regulation by the *LIN28*/*let-7* pathway does not require stem cell reprogramming to increase fetal hemoglobin expression [2, 36]. Studies are now being focused upon erythroid-specific features of LIN28 expression with particular interest upon identifying a mechanistic bridge between the *LIN28*/*let-7* pathway and globin gene regulation. Our results may also be applied toward topics of globin gene therapy where erythroid-specific expression may be advantageous for safety concerns as well as therapeutic effects.

## Supporting Information

S1 File DNA sequence of human KLF1 Promoter and SPTA1 Promoter for construction of the KLF1-LIN28A-OE and SPTA1-LIN28A-OE lentiviral expression vector. (DOCX)
