# Supplementary material for: Erythroid-Specific Expression of LIN28A Is Sufficient for Robust Gamma-Globin Gene and Protein Expression in Adult Erythroblasts
Source: PLoS One. 2015 Dec 16;10(12):e0144977. doi: 10.1371/journal.pone.0144977 (PMC4684222; doi:10.1371/journal.pone.0144977)
Supplement: S1 File — (DOCX) [file pone.0144977.s001.docx]

**Lee et. al., S1 File**

**S1 File.** DNA sequence of human KLF1 Promoter and SPTA1 Promoter used for construction of the KLF1-LIN28A-OE and SPTA1-LIN28A-OE lentiviral expression vector.

>KLF1 Promoter Sequence (1056 bp)

GGCTGGTCTTGAAATCCTGGTGTCAAGTGATCCTCCTGCCTCAGTCTTCCAATGAGTGACTATGAGTTATGTGTCTGTAATACGTATCCATGTCCCCTTCCCAGGCTCCCAGGGCACCAGAGTGGAGGTTCCTGTTGTAGAAACTCAGATCCTCTCCTCATGTTGGGCAGAATCAAGGAGCAGCCAGGCCCAGAGCCAGGGCACTGGTCTCCTGCAGGTCAGGTACTGCCTGTGGCTTGATCCAACGGTCCTATCCCACCCAGGAGGAGAGAGGGTCACTTTTCCCTTGGCTGCCCCCATCGCACTAAAGCAGCTGGCACTGAACCAGCCTCCATGCAGTCCCATGCAGTGCCACCCAAGGGTCCCCAGTAGACAATGGTGGGCCAGTTGTCAGGGGCTTCCTCCTGCTGCAGGGCTGAGACCCTGGGAGGTCCCAACCCAGGCAAATTGAACGCCAGGCTAATTTGAAGACCCAACTCCCAGCCCTCCCCTTCACCGGAGGACAGAGCTCTAGCTGGCCTGGGCCCCCACCTGATAGCAGCCTCCAACGTCTGGGGTGTCTGATAATGCTTGGCGGGGAGCTCGTGCCAAGTCCCGCCATCAGCACGGTTGTTGCTGTTTACTGGGGAGGGGGAGGGCTGTGGAGCCTCAATCAGGGGGACAGGGGGTCCCACAGCTTCTTCCCAGAATACCCTTTCTGCCTTTTCCAGGAAAGTTAACTGAGGGAAGACCCCCAAGTCTCTCCTTCTTTGGAGACCCAATGTCTGTTTTTACCCAGCACCTGGACCCTCAAACCCTGAACCCCCCAACCCTTGATATTTGACTTGGCTTTGGACACAGGGTTAGTCTTTAACCCCAGCCCCAGACAGGCCAACGTGAAGTTTGTGCCCCAGAAACAGTGCCCCCCCGCCGCCTTGCCTTGCTTTGCCTTATCAGAGGCTGCAGCCAATCAGCTAAGGACAGAGAGGAGCCCTCGAAGGGGCTATCACAGCCTCAGAGTTCACGAGGCAGCCGAGGAAGAGGAGGCTTGAGGCCCAGGGTGGGCACCAGCCAGCC

>SPTA1 Promoter Sequence (966 bp)

CCAGACTTTCAAGAAGAGAATGTAAAGGACTCTCAGTTTTTCTATGTGAGATCCAAGAACAGGGGAAACCACAAATGTAGAATCAAAATCTTCTCTTGTAGATCATGGAAGTTAGATTATTCAAGCTGCATTTACACTGGAATGAAGTTTGGCATGGATAGCCCCACTGGCTTGTAGAAAGGTCTAAGAGCATCCCACTATTTTCTTTTTGCTTGTTTGTTTCATTTTTGTAAGTTAGTCTAAAATAACTATGCCTATGGAAAAAGTCTACCTCTTTTTGTTCTCCATTAAAATTAATACATACAGTAGGATGATTTATACCATATATATCAGAAGTCAGCCATATTTTCAACTTTTTTGTGACATCATTGGTTTGTCAAATAAGGTATCCTAATTTTTGTTTTACAAAATATTAATGAACACATCATATAACTTTTTTTGTTCCTTCCATTTAAATGATTAAGAGTACAACTAAATCAGATTTTTAAAGACCAAATTGTTGAGGTTAGGAATAAAATCTTATTTTGTGAAAGACTCTCTAGAAAAAGCCAGGATAATTCGTTGACTTAAAGACTGTGAAAAATCTGCAGTTGAGTTGGATGAAGTTGTTGAGCTGTATTTCCTATAAATATATGGGGTTTTTTTTCTAATTTTTCATCCAAAAACCTTACTTTCATTTTTTAAGTCTTATTGAGTAACTTTGAAAGCCAGAGCCTCCCAAAACTGCTGAGTCACCCAGTATCTGTAAAACTTAGCAGTTGCCTCAGCTGAGTATGTCTTCTAAAGATAATGTCGATTGTGTATGGCTGATGGGATTCTAGGACCAAGCAAGAGGTTTTTTTTTTTCCCCCACATACTTAACGTTTCTATATTTCTATTTGAATTCGACTGGACAGTTCCATTTGAATTATTTCTCTCTCTCTCTCTCTCtctcTGACACATTTTATCTTGCCAGGTTCTAAACCC
